# Multiple statistical models reveal specific volatile organic compounds affect sex hormones in American adult male: NHANES 2013–2016

**DOI:** 10.3389/fendo.2022.1076664

**Published:** 2023-01-12

**Authors:** Chengcheng Wei, Li Cao, Yuancheng Zhou, Wenting Zhang, Pu Zhang, Miao Wang, Ming Xiong, Changqi Deng, Qi Xiong, Weihui Liu, Qingliu He, Yihong Guo, Zengwu Shao, Xiaogang Chen, Zhaohui Chen

**Affiliations:** ^1^ Department of Urology, Union Hospital, Tongji Medical College, Huazhong University of Science and Technology, Wuhan, Hubei, China; ^2^ Department of Orthopaedic, Union Hospital, Tongji Medical College, Huazhong University of Science and Technology, Wuhan, Hubei, China; ^3^ Department of Obstetrics and Gynecology, Union Hospital, Tongji Medical College, Huazhong University of Science and Technology, Wuhan, Hubei, China; ^4^ Chongqing Medical University, Chongqing, China; ^5^ Department of Urology, The Second Affiliated Hospital of Fujian Medical University, Quanzhou, China; ^6^ Department of Urology, Huangshi Central Hospital, The Affliated Hospital of Hubei Polytechnic University, Huangshi, China

**Keywords:** volatile organic compounds (VOCs), testosterone (TT), estradiol (E2), SHBG, machine learning

## Abstract

**Background:**

Some VOCs are identified as endocrine-disrupting chemicals (EDCs), interfering with the effect of sex hormones. However, no studies focused on the common spectrum of environmental VOCs exposure affecting sex hormones in the average male population.

**Objectives:**

We aimed to explore the association between VOCs and sex hormones in American adult males using multiple statistical models.

**Methods:**

The generalized linear (GLM), eXtreme Gradient Boosting (XGBoost), weighted quantile sum (WQS), Bayesian kernel machine regression (BKMR) and stratified models were used to evaluate the associations between Specific Volatile Organic Compounds and sex hormones in American adult male from NHANES 2013–2016.

**Results:**

Pearson correlation model revealed the potential co-exposure pattern among VOCs. XGBoost algorithm models and the WQS model suggested the relative importance of VOCs. BKMR models reveal that co-exposure to the VOCs was associated with increased Testosterone (TT), Estradiol (E_2_), SHBG and decreased TT/E_2_. GLM models revealed specific VOC exposure as an independent risk factor causing male sex hormones disorders. Stratified analysis identified the high-risk group on the VOCs exposures. We found Blood 2,5-Dimethylfuran in VOCs was the most significant effect on sex hormones in male. Testosterone increased by 213.594 (ng/dL) (124.552, 302.636) and estradiol increased by 7.229 (pg/mL) for each additional unit of blood 2,5-Dimethylfuran (ng/mL).

**Conclusion:**

This study is an academic illustration of the association between VOCs exposure and sex hormones, suggesting that exposure to VOCs might be associated with sex hormone metabolic disorder in American adult males.

## 1 Introduction

Volatile organic compounds (VOCs) are a broad class of chemicals. Apart from natural sources (1), many VOCs are produced by anthropogenic activities, including fuel combustion, petroleum refining, vehicle emissions, and chemical processes in the industry (manufacturing of paints, solvents, and other oil derivatives) (2, 3). Since these compounds can volatilize, dissolve in water and adhere to particles, people absorb them unconsciously by inhalation, dermal contact, and consuming of polluted water and food (2, 4). The amount of exposure to VOCs is assessed by monitoring their concentration in the relevant space or by measuring the quantity of VOCs and their metabolites in blood and urine (5).

A considerable amount of literature has been published on various aspects of health hazards caused by exposure to VOCs. A study demonstrated that VOCs showed significant toxicity to mouse testis by altering testosterone levels and testicular marker enzyme activity. Song has reported DNA damage to the spermatic cord for two years in 27 workers exposed to benzene (86.49±2.83mg/m) (6). Similarly, low sperm motility was noted in 50 aircraft maintenance workers exposed to benzene containing jet fuel (7). However, the effect of VOCs on human sex hormones has not been clarified.

Sex hormones are essential for the development and function of the reproductive system. Total testosterone (TT) and estradiol (E_2_) are two main critical sex hormones in the human body. TT plays a key role in the differentiation of the reproductive system, spermatogenesis, and development of secondary sexual characteristics in males. Similarly, E_2_ promotes female sexual development and stimulates the maturation of primary and secondary sex characteristics in females (8). Estrogen is of significance to males as well, regulating bone formation, nutrient metabolism and reproductive function (9). In contrast, Sex hormone-binding globulins (SHBGs) are a group of transporter proteins in plasma that bind and transport testosterone and estradiol, regulating the concentration of non-protein-bound sex hormones recognized as biologically active (10).

Benzene, toluene, ethylbenzene, and xylene are identified as endocrine-disrupting chemicals (EDCs), interfering with the effect of sex hormones (11, 12). However, till now, there have been nearly no studies focused on the common spectrum of environmental VOCs affecting the levels of sex hormones in the average male population. We hypothesized that VOCs might contribute to reproductive toxicity, which leads to changes in sex hormone concentration. In order to verify our hypothesis, we explored the U.S. National Health and Nutrition Examination Survey (NHANES) for secondary analysis. We controlled the potential confounders, including age, race, education level, marital status, poverty to income ratio, BMI, alcohol drinks, and smoking which might be related to both the exposure as well as the outcome. Moreover, we constructed machine learning of XGBoost algorithm models, Weighted Quantile Sum (WQS) regression, generalized linear (GLM) and stratified analysis to explore the relative importance of selected VOCs on sex hormones. Furthermore, we implemented Bayesian kernel machine regression (BKMR) to identify the overall effect of the eight specific blood VOCs exposure on every single sex hormone. We aim to illustrate the VOCs’ influence on reproductive health among U.S. males, helping humans avoid impaired reproductive VOCs.

## 2 Methods

### 2.1 Availability of data

The National Health and Nutrition Examination Survey (NHANES) is a large-scale cross-sectional survey in the United States collecting citizens’ personal and health-relevant data through interviews, examinations and laboratory tests. It has been conducted to assess Americans’ health and nutritional status since the early 1960s under the guidance of the National Center for Health Statistics (NCHS) and the Centers for Disease Control and Prevention (CDC). All these data and the process of data collecting and measurement are available for the public on its official website. Since NHANES processes were permitted by the NAHNES Institutional Review Board (IRB)/NCHS Research Ethics Review Board (ERB), there are no additional approvals required for our study (13).

### 2.2 Study population

**Figure 1:**
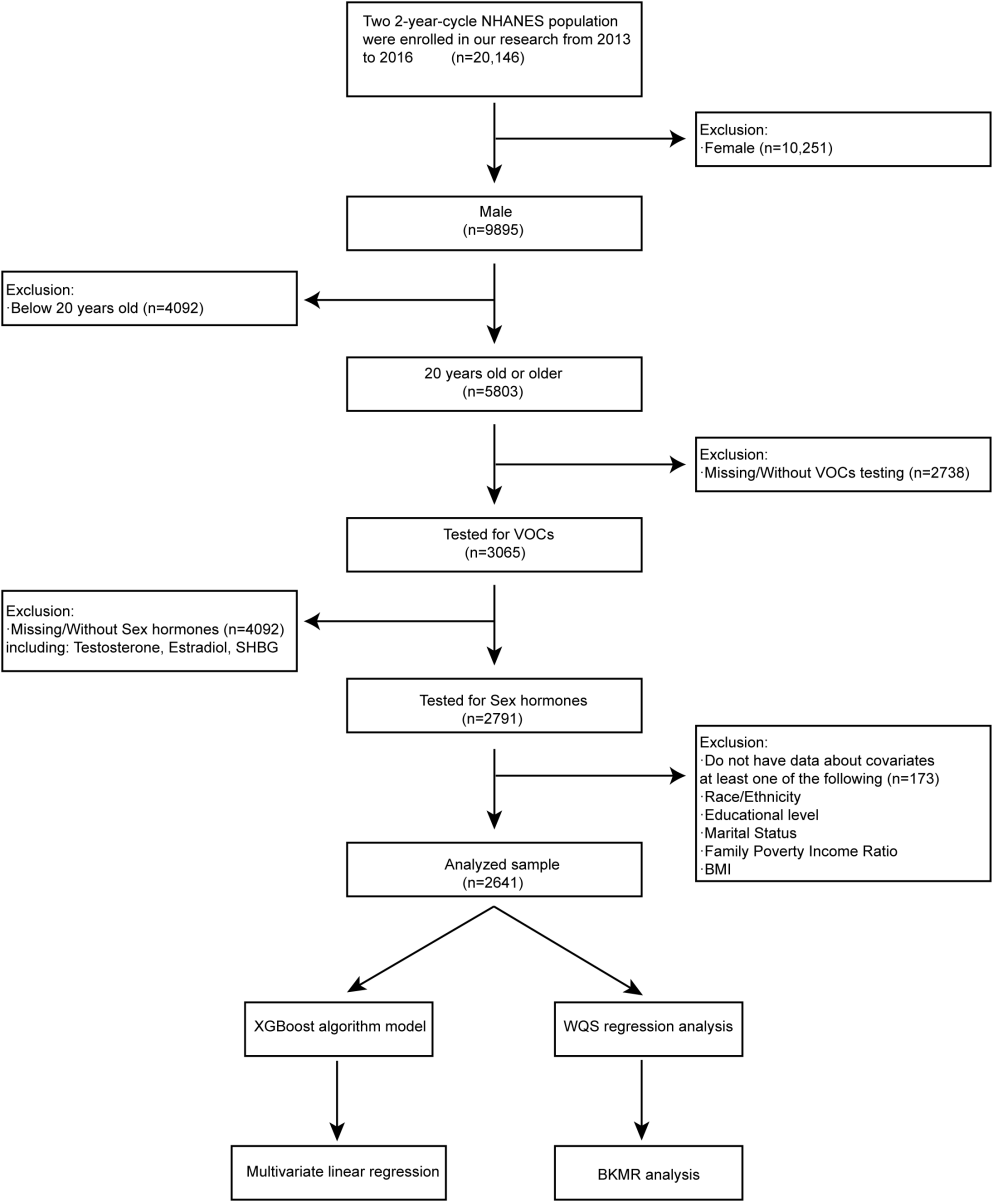
Flowchart of studied participants selection (N=2641) and analysis process, NHANES, USA, 2013-2016.

NHANES released its data in two-year cycles since 1999. Our study included participants from the latest two cycles, 2013-2016, whose relevant data was available on the NHANES website. Owing to lacking the complete results of sex hormones measurement, the data before 2013 were excluded. Sociodemographic data, comorbidities data, medical examination, and personal life history data, as well as laboratory data of sex hormone concentrations and blood VOCs, have been included in our study for the secondary analysis. To be eligible for analysis, participants had to meet the following inclusion criteria: (1) male subjects (n = 9,895); (2) 20 years old or older (n = 5,803); (3) Tested for VOCs (n = 3,065); (4) Tested for sex hormones (n = 2,791); (5) have data about covariates of following (n = 2,641): race/ethnicity; educational level; marital level; family poverty income ratio; body mass index (14–16). The exclusion criteria were as follows: (1) female subjects (n = 10,251); (2) aged below 20 years old (n = 4,092); (3) missing/without VOCs testing (n = 2,738); (4) missing/without sex hormones results including Testosterone, Estradiol and SHBG (n = 4,092); (5) do not have data about covariates at least one of following (n = 173): race/ethnicity; educational level; marital level; family poverty income ratio; body mass index. There were 2,641 participants out of 20,146 left in the analysis after selecting and excluding according to the criteria mentioned above (**Figure 1**). In addition, the whole process of our study complied with the Helsinki Declaration of the World Medical Association (17).

### 2.3 Sex hormones measurement

After dissociation from binding proteins and removal of potentially interfering compounds, the total amount of testosterone and estradiol in serum are determined by isotope dilution high-performance liquid chromatography-tandem mass spectrometry (ID-LC-MS/MS) using stable isotope-labelled internal standards and external calibrators. Subsequent to the reaction of SHBG with immuno-antibodies and exerting a magnetic which leads to the capture of microparticles on an electrode, SHBG can be gauged indirectly by a chemiluminescent measurement *via* photomultiplier tube. Testosterone/Estradiol was used to estimate the proportion of the sex hormones. The details of measurement can be found in the laboratory procedure manual on the NHANES website.

### 2.4 VOCs measurement

VOCs are widely used in industry and daily life. Biomonitoring of blood VOCs provides valuable information on exposure and their internal dose. Volatile organic compounds are measured in especially collected whole blood samples by headspace solid-phase microextraction (SPME)/gas chromatography/isotope dilution mass spectrometry. This method is efficient for quantifying a broad range of VOCs in a small amount of blood. Furthermore, it is also suitable for detecting blood VOCs of the average individuals for its shallow detection limit. By minimizing the sources of contamination, maintaining laboratory performance and retesting 2% of all specimens randomly, the quality of data was guaranteed. In our research, we included the main VOCs exposures, including Blood 2,5-Dimethylfuran, Blood Tetrachloroethene, Blood Benzene, Blood 1,4-Dichlorobenzene, Blood Ethl Acetate, Blood Furan, Blood Toluene and Blood m-/p-Xylene (14). For VOCs, if those analytical results are below the lower limit of detection, an estimated fill-in value that is the lower limit of detection divided by the square root of two will be put in the analytical results.

### 2.5 Other variables

Based on previous studies, other variables which may affect sex hormone concentrations were included in our study as well. In detail, sociodemographic variables contained age (year), poverty to income ratio, race/ethnicity (Mexican American, other Hispanic, non-Hispanic white, non-Hispanic black, others), level of education (less than 9th grade, 9-11th grade, high school graduate, AA degree, college graduate, don’t know), marital status (married, widowed, divorced, separated, never married, living with a partner, don’t know). Moreover, selected comorbidities data comprised drinking (Had at least 12 alcohol drinks/1 year) and smoking (Smoked at least 100 cigarettes in life). Last, we also introduced body mass index (Kg/m2) as medical examination and personal life history data. More complex variables can be found on the NHANES official website (18).

### 2.6 Statistical analysis

Adhering to the CDC guidelines’ criteria, we conducted a statistical analysis of the serum VOCs and sex hormone levels (https://www.cdc.gov/nchs/nhanes/index.htm). The analytic guidelines from NHANES were referred for statistical analysis (https://wwwn.cdc.gov/nchs/nhanes/tutorials/default2.aspx). For weighted analysis, we utilized the subsample weights provided in the VOCs sample. The continuous variables, such as VOCs and sex hormone concentrations, were described by the normal distribution with mean μ and standard deviation σ. The categorical variables were presented as a percentage or frequency.

First, the participants were divided into three terciles based on age as a continuous variable. We applied the Kruskal Wallis rank sum test to calculate the p-value of the continuous variables whose theoretical number is greater than or equal to 10. While for those continuous variables having a theoretical number < 10, Fisher’s exact probability test was employed to calculate the p-value. In the case of categorical variables, we utilized the weighted chi-square to calculate the p-value. Results are shown in **Table 1**. Second, we set up a Pearson correlation model among specific VOCs to investigate potential co-exposure patterns and co-toxicity effects. Then, we constructed the XGBoost algorithm model (19)of machine learning to explore the relative importance of influence that serum VOCs exert on sex hormone concentrations by analyzing the contribution (gain) of sex hormone concentrations that VOCs brought (20). Furthermore, the Weighted Quantile Sum (WQS) regression model (21) was utilized to evaluate the weights of each specific blood VOCs on every single sex hormone with adjustment or not. The WQS index was calculated by dividing eight kinds of VOCs into quartiles, and the weight of each VOCs was estimated by bootstrapping (n = 1000). Moreover, we built three kinds of multivariate weighted linear model analysis between VOCs and sex hormone concentrations with different ranges of adjustment to variables to further clarify the relationship between the two. The generalized linear (GLM) results were based on Rubin’s rules and calculated dataset. Last, we implemented Bayesian kernel machine regression (BKMR) to identify the overall effect of the eight specific blood VOCs exposure on every single sex hormone (22). The BKMR model underwent 20,000 iterations of the Markov chain Monte Carlo technique. Besides, we investigated the univariate exposure-response relationship between each log-transformed VOCs concentration and estimates of sex hormone with all other VOCs held at their 50th percentiles. We further constructed the subgroup analysis to identify the stratified associations between blood VOCs and sex hormones through stratified multivariate logistic regression.

**Table 1:** Baseline characteristics of selected participants.

Exposure	Low Exposure*	High Exposure*	
N	1320	1321	
Sociodemographic variables			
Age, mean±SD (years)	49.863 ± 18.051	48.339 ± 16.078	0.022
Poverty to income ratio, mean±SD	2.789 ± 1.594	2.123 ± 1.417	<0.001
Race/Ethnicity (%)			<0.001
Mexican American	189 (14.318%)	181 (13.702%)	
Other Hispanic	141 (10.682%)	134 (10.144%)	
Non-Hispanic White	566 (42.879%)	482 (36.488%)	
Non-Hispanic Black	184 (13.939%)	357 (27.025%)	
Other race/ethnicity	240 (18.182%)	167 (12.642%)	
Education (%)			<0.001
Less than 9th grade	117 (8.864%)	161 (12.188%)	
9-11th grade	124 (9.394%)	245 (18.547%)	
High school graduate	264 (20.000%)	384 (29.069%)	
AA degree	361 (27.348%)	363 (27.479%)	
College graduate	454 (34.394%)	166 (12.566%)	
Don’t know	0 (0.000%)	2 (0.151%)	
Marital status (%)			<0.001
Married	798 (60.455%)	654 (49.508%)	
Widowed	45 (3.409%)	35 (2.650%)	
Divorced	94 (7.121%)	159 (12.036%)	
Seperated	14 (1.061%)	58 (4.391%)	
Never married	264 (20.000%)	257 (19.455%)	
Living with partner	104 (7.879%)	158 (11.961%)	
Don’t know	1 (0.076%)	0 (0.000%)	
**Medical examination and personal life history**			<0.001
Body mass index, mean ± SD (Kg/m2)	29.055 ± 6.258	27.954 ± 5.988	
Comorbidities (%)			
Had at least 12 alcohol drinks/1 year?			<0.001
Yes	1008 (76.364%)	1098 (83.119%)	
No	312 (23.636%)	223 (16.881%)	
Smoked at least 100 cigarettes in life			<0.001
Yes	555 (42.045%)	1002 (75.852%)	
No	764 (57.879%)	317 (23.997%)	
Sex hormones			
Testosterone, mean ± SD (ng/dL)	402.229 ± 163.278	448.004 ± 202.493	<0.001
Estradiol, mean ± SD (pg/mL)	24.808 ± 9.655	24.605 ± 9.789	0.591
SHBG, mean ± SD (nmol/L)	43.391 ± 24.753	48.290 ± 28.113	<0.001
Testosterone/Estradiol, mean ± SD	14.346 ± 10.403	14.953 ± 8.393	0.099
**Blood VOCS**			
Blood 2,5-Dimethylfuran, mean (SD) Median (Min-Max) (ng/mL)	0.009 (0.005) 0.008 (0.008-0.057)	0.089 (0.112) 0.055 (0.008-1.450)	<0.001
Blood Tetrachloroethene, mean (SD) Median (Min-Max) (ng/mL)	0.039 (0.021) 0.034 (0.034-0.321)	0.120 (0.827) 0.034 (0.034-16.000)	<0.001
Blood Benzene, mean (SD) Median (Min-Max) (ng/mL)	0.024 (0.020) 0.017 (0.017-0.285)	0.178 (0.273) 0.109 (0.017-6.290)	<0.001
Blood 1,4-Dichlorobenzene, mean (SD) Median (Min-Max) (ng/mL)	0.057 (0.055) 0.028 (0.028-0.372)	1.706 (7.329) 0.085 (0.028-115.000)	<0.001
Blood Ethl Acetate, mean (SD) Median (Min-Max) (ng/mL)	0.116 (0.034) 0.112 (0.112-0.407)	0.699 (3.920) 0.112 (0.112-62.300)	<0.001
Blood Furan, mean (SD) Median (Min-Max) (ng/mL)	0.018 (0.002) 0.018 (0.018-0.078)	0.056 (0.057) 0.033 (0.018-0.605)	<0.001
Blood Toluene, mean (SD) Median (Min-Max) (ng/mL)	0.074 (0.047) 0.060 (0.018-0.317)	0.518 (0.794) 0.340 (0.018-14.700)	<0.001
Blood m-/p-Xylene, mean (SD) Median (Min-Max) (ng/mL)	0.049 (0.034) 0.040 (0.024-0.305)	0.284 (0.907) 0.163 (0.024-19.600)	<0.001

Our data included Sex hormones, Blood VOCs, Sociodemographic variables, Medical examination and personal life history data and Comorbidities for the secondary analysis.

*We identify the summary of VOCs as the Total exposure and group them into second classification including low exposure and high exposure.

Results showed that there exists no significant difference between the complete data and the original data. Overall, multiple analysis results were based on the calculated dataset as well as Rubin’s rules. All kinds of statistical analyses were conducted by R software (Version 4.0.2) using the R package (http://www.R-project.org, The R Foundation) (23). The software of EmpowerStats provided significant help in the process of our research (http://www.empowerstats.com, X&Y Solutions, Inc., Boston, MA, USA). In our study, it is considered to be of statistical significance when the p-value is less than 0.05.

## 3 Results

### 3.1 Baseline characteristics of selected participants, NHANES, USA, 2013–2016

The baseline characteristics of American adult males from 2013 to 2016 were presented in **Table 1**, which grouped the population by two groups respectively according to the exposure of VOCs as well as weighted distribution. We identify the summary of VOCs as the total exposure and group them into second classification including low exposure and high exposure. A total of 2641 participants were included in our study. Variables included sociodemographic variables, comorbidities, medical examination and personal life history, sex hormones and blood VOCs. The distribution of eight specific VOCs exposure showed statistical difference compared with two exposure groups. Among male sex hormones in various exposure groups, we found the distribution of testosterone and SHBG indicated statistical difference with p values < 0.05, estradiol and TT/E_2_ showed no statistical difference with p values > 0.05. Interestingly, the distribution of age, poverty to income ratio, race, education level, marital status showed statistical difference compared with two exposure groups (**Table 1**).

### 3.2 Correlation analysis among specific VOCs

**Figure 2:**
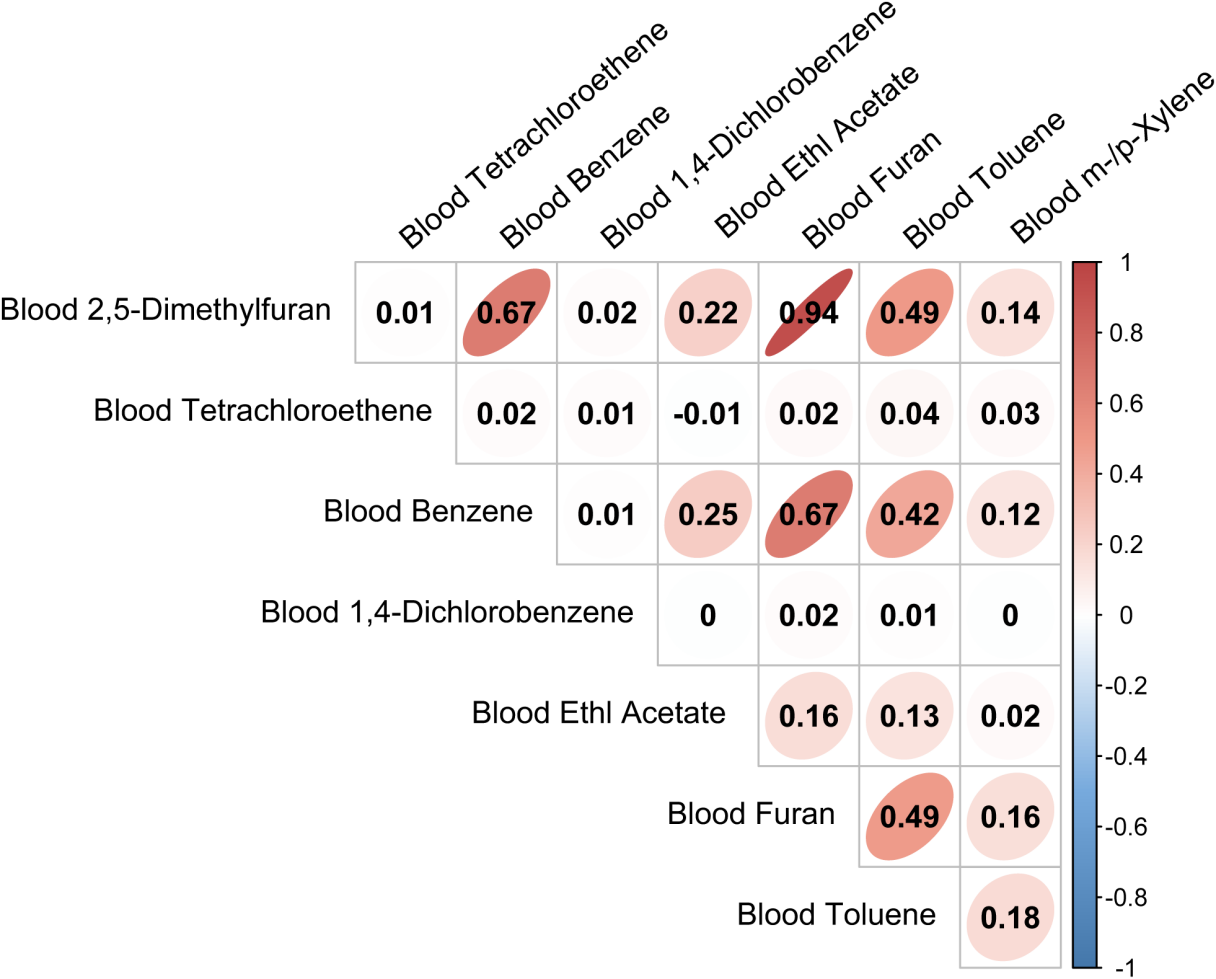
Heatmap of Pearson correlation analysis among specific VOCs.

In order to explore the potential co-exposure pattern, we have constructed a Pearson correlation model among specific VOCs, and we presented the results as the heatmap in (**Figure 2**). We found Blood 2,5-Dimethylfuran was strongly correlated with Blood Furan (r=0.94). Meanwhile, Blood Benzene was strongly correlated with 2,5-Dimethylfuran (r=0.67) and Blood Furan (r=0.67). Strong correlations may indicate co-exposure or co-toxicity effect. Other correlations were not significant among these VOCs.

### 3.3 XGBoost algorithm models reveal the relative importance of VOCs on sex hormone

**Figure 3:**
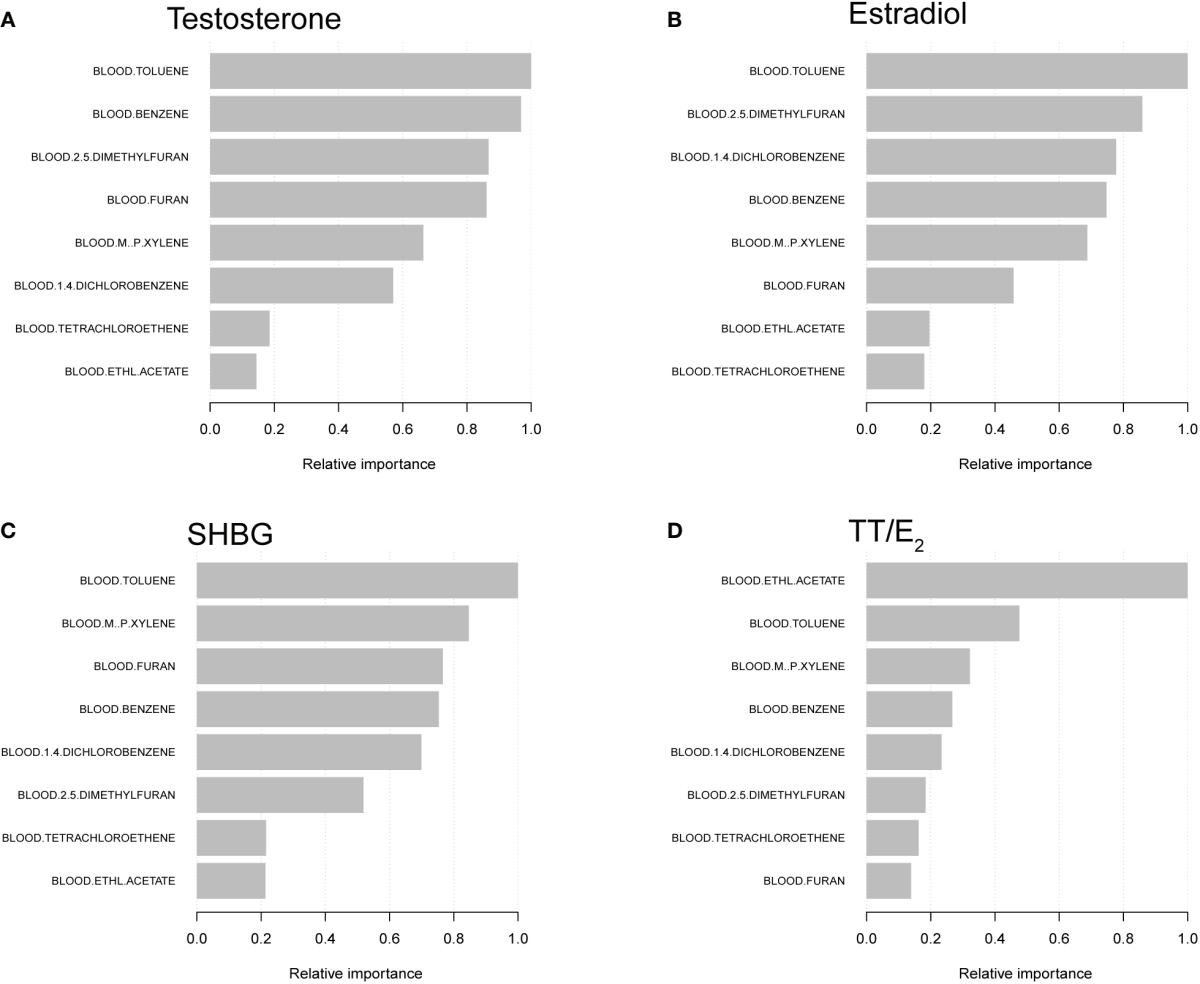
XGBoost models indicate the relative importance of specific blood VOCs on sex hormones and the corresponding variable importance score. **(A)** The relationship between testosterone and VOCs. **(B)** The relationship between estradiol and VOCs. **(C)** The relationship between SHBG and VOCs. **(D)** The relationship between the TT/E_2_ and VOCs. The X-axis indicates the importance score, the relative number of a variable used to distribute the data; the Y-axis shows the specific blood VOC.

We further constructed machine learning of XGBoost algorithm models to explore the relative importance of selected VOCs on sex hormones. We found that blood Toluene was the most critical variable in testosterone, followed by blood Benzene, blood 2,5-Dimethylfuran, blood Furan and blood m-/p-Xylene. For the estradiol, the order of relative importance was blood Toluene, blood 2,5-Dimethylfuran, blood 1,4-Dichlorobenzene, blood Benzene and blood m-/p-Xylene. For the SHBG, blood Toluene was the most important factor, followed by blood m-/p-Xylene, blood Furan, blood Benzene and blood 1,4-Dichlorobenzene. Furthermore, blood Ethl Acetate was the most critical variable on TT/E_2_ (**Figure 3**).

### 3.4 WQS regression analysis of associations between VOCs and sex hormones

**Figure 4:**
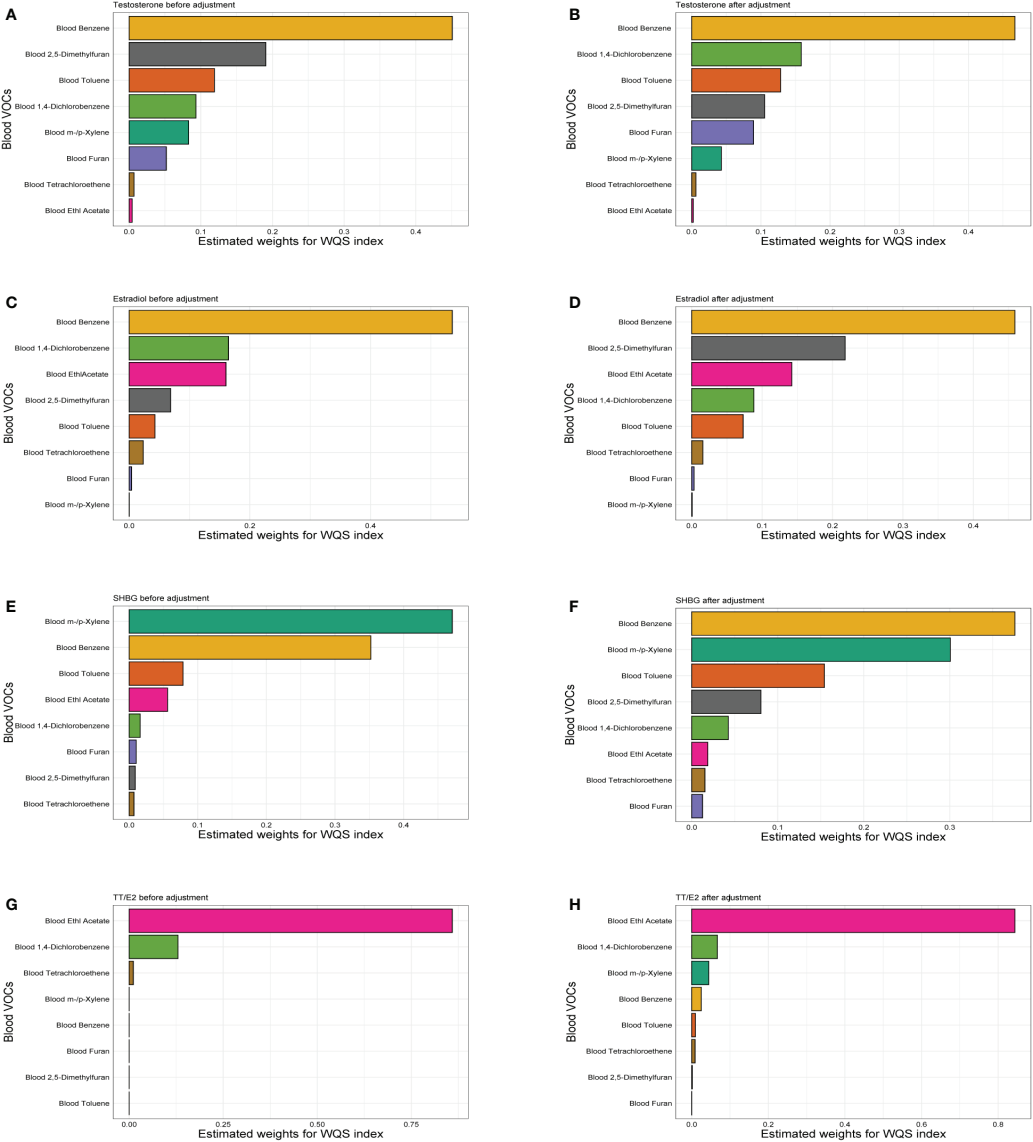
The WQS regression model estimated weights of each specific blood VOCs associated with sex hormones. **(A)** Weights in the WQS index containing specific blood VOCs in the models of male testosterone before adjustment. **(B)** Weights in the WQS index containing specific blood VOCs in the models of male testosterone adjust for age, race, education, marital status, body mass index, race, family income to poverty ratio, smoking and drinking. **(C)** WQS model of VOCs and male estradiol before adjustment. **(D)** WQS model of VOCs and male estradiol after adjustment. **(E)** WQS model of VOCs and male SHBG before adjustment. **(F)** WQS model of VOCs and male SHBG after adjustment. **(G)** WQS model of VOCs and male TT/E2 before adjustment. **(H)** WQS model of VOCs and male TT/E2 before adjustment.

We constructed WQS regression to analyze the possible association between selected VOCs exposure and sex hormones (**Figure 4**). Results showed positive associations between all VOCs and testosterone, estradiol and the SHBG. A negative relationship existed between VOCs and TT/E_2_. After adjustment, we found the VOCs of the largest weight in testosterone, Estradiol and the SHBG effect was blood Benzene. For the testosterone, estimated weight followed by blood 1,4-Dichlorobenzene, blood Toluene. For the estradiol, the estimated weight was followed by blood 2,5-Dimethylfuran and blood Ethl Acetate. For the SHBG, the estimated weight was followed by blood m-/p-Xylene and blood Toluene. Moreover, Blood Ethl Acetate was the largest weight in TT/E_2_. Weight quantification of WQS analysis was in the (**Supplementary Table 1**).

### 3.5 Independent associations between VOCs exposure and sex hormones

**Figure 5:**
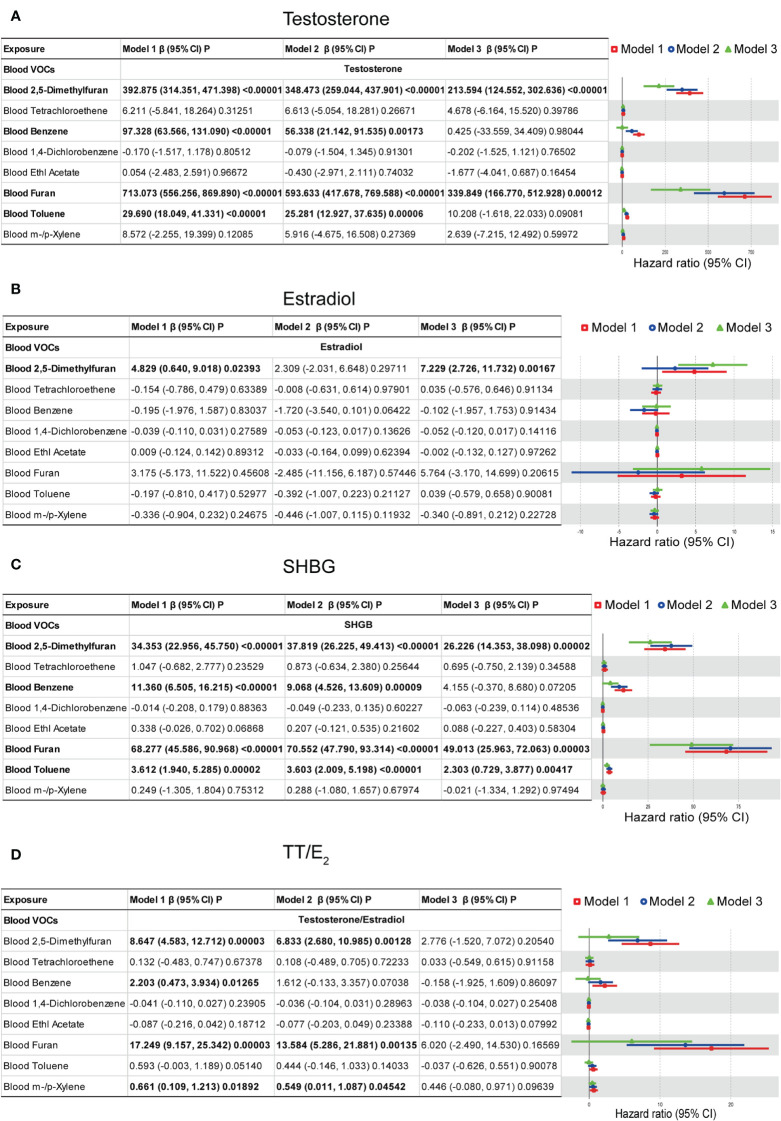
Multivariate weighted linear model analysis between VOCs and sex hormones. **(A)** Models of blood VOCs and male testosterone. **(B)** Models of blood VOCs and male estradiol. **(C)** Models of blood VOCs and male SHBG. **(D)** Models of blood VOCs and male TT/E2. Model 1: adjusts for none. Model 2: Minimally adjusted model which adjusts for age, race/ethnicity, education level, poverty income ratio, and marital status. Model 3: Fully adjusted model which adjusts for age, race, education, marital status, body mass index (kg/m2), family income to poverty ratio, smoking and drinking.

The generalized linear (GLM) models were used to explore the association between the blood VOCs and sex hormones which is shown in **Figure 5**. For the sex hormones of testosterone, we found that blood 2,5-Dimethylfuran, blood Benzene, blood Furan, and blood Toluene shows positive associations with statistical significance. In the model 3 (fully adjusted model), results indicated that testosterone increased by 213.594 (ng/dL) (124.552, 302.636) for each additional unit of blood 2,5-Dimethylfuran (ng/mL), meanwhile, increased by 339.849 (ng/dL) (166.770, 520.928) for each additional unit of blood Furan (ng/mL). For the estradiol, we found that only blood 2,5-Dimethylfuran shows a positive association with statistical significance. In model 3 (fully adjusted model), results indicated that estradiol increased by 7.229 (pg/mL) (2.726, 11.732) for each additional unit of blood 2,5-Dimethylfuran (ng/mL). For the SHBG, blood 2,5-Dimethylfuran, blood Furan and blood Toluene show positive associations with statistical significance. In model 3 (fully adjusted model), results indicated that SHBG increased by 26.226 (nmol/L) (14.353, 38.098) for each additional unit of blood 2,5-Dimethylfuran (ng/mL), increased by 49.013 (nmol/L) (25.963, 72.063) for each additional unit of blood Furan (ng/mL) and increased by 2.303 (ng/dL) (0.729, 3.877) for each additional unit of blood Toluene (ng/mL). Multivariate regression models show no association between TT/E2 and VOCs with statistical significance. These results suggested that long-time environmental VOCs exposure as an independent risk factor may cause males metabolic disorders damage, especially in the male reproductive gland.

### 3.6 The association between VOCs and sex hormones by BKMR analysis

**Figure 6:**
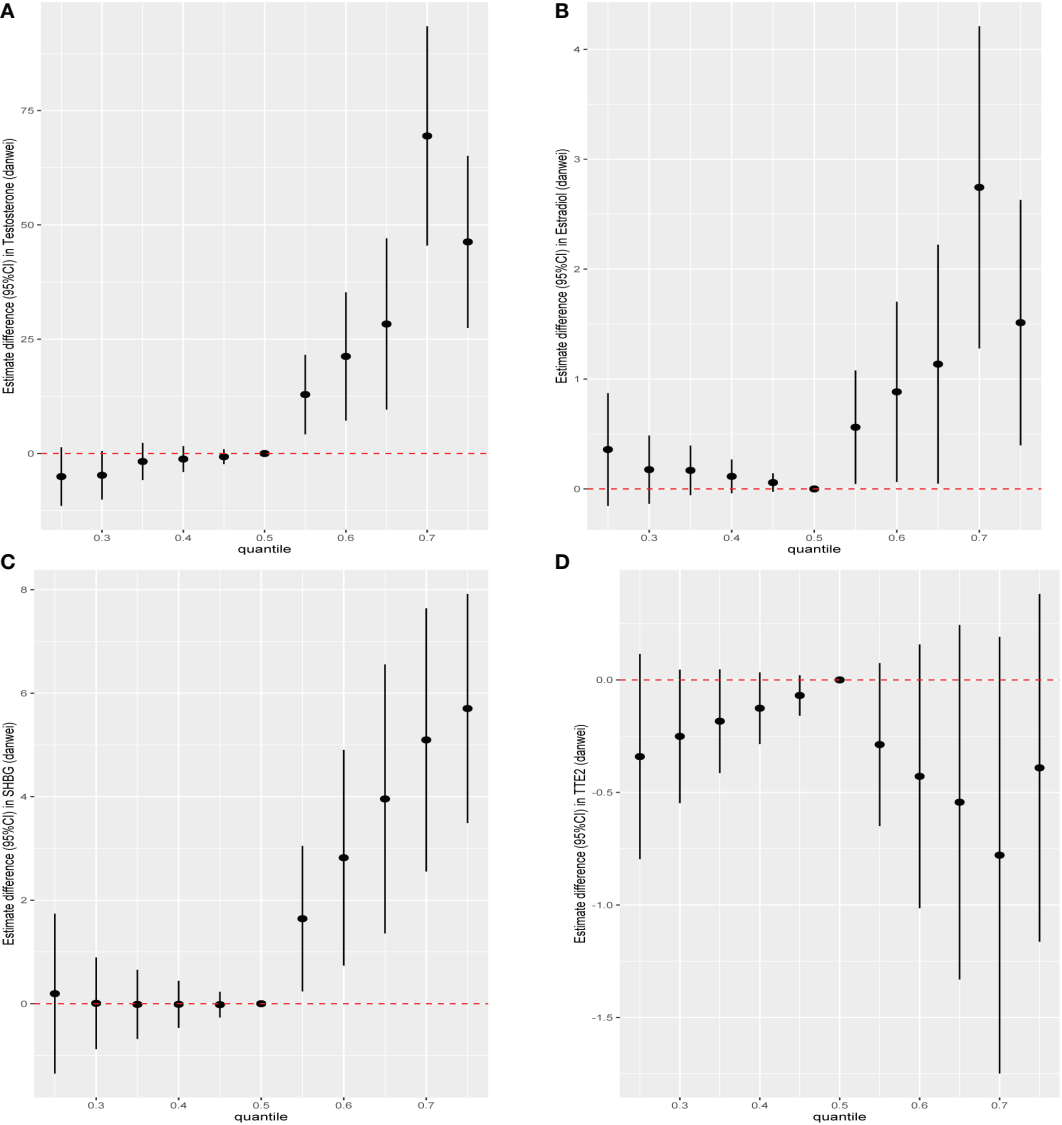
The overall effect of the eight specific blood VOCs exposure on **(A)**male testosterone, **(B)** male estradiol, **(C)** male SHBG and **(D)** TT/E2 using the BKMR model. The vertical ordinate showed the estimated change in sex hormones in relation to the eight specific blood VOCs at a particular percentile (labelled at the X-axis) compared to corresponding medians.

**Figure 7:**
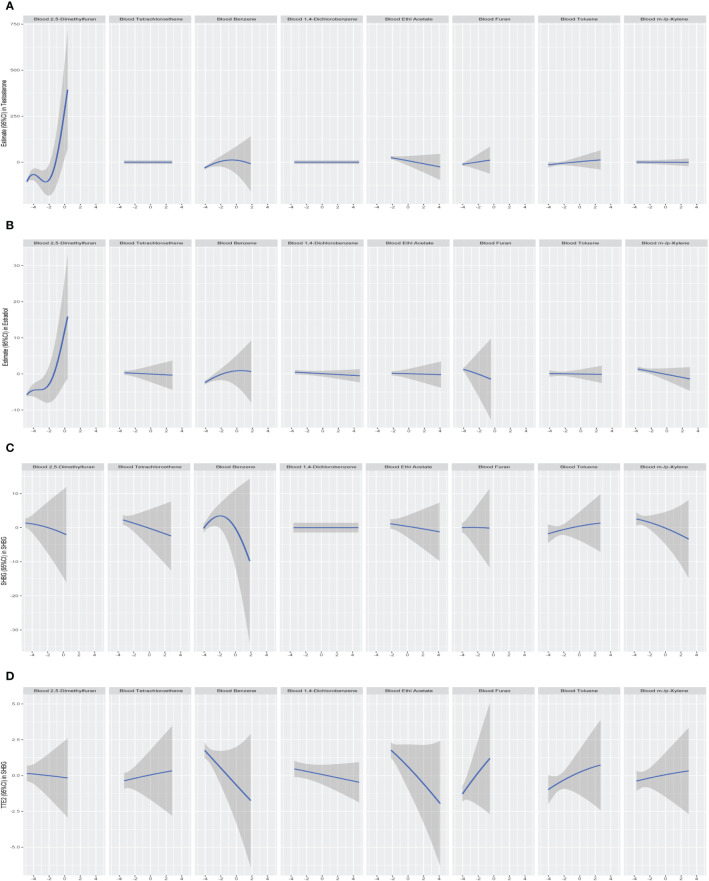
Univariate exposure-response relationship between each log-transformed VOCs concentration and estimates of sex hormone all other VOCs were held at their 50th percentiles. **(A)** Models of blood VOCs and male testosterone. **(B)** Models of blood VOCs and male estradiol. **(C)** Models of blood VOCs and male SHBG. **(D)** Models of blood VOCs and male TT/E_2_. Models adjusted for age, race, education, marital status, body mass index (kg/m2), family income to poverty ratio, smoking and drinking.

**Figure 8:**
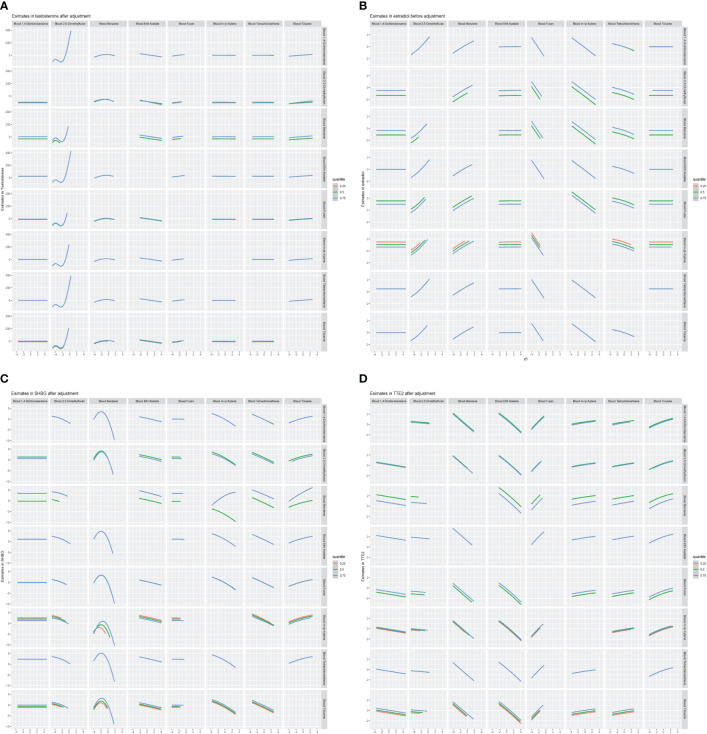
Bivariate exposure-response relationship between VOCs and sex hormones. **(A)** Models of blood VOCs and male testosterone. **(B)** Models of blood VOCs and male estradiol. **(C)** Models of blood VOCs and male SHBG. **(D)** Models of blood VOCs and male TT/E2. Each VOCS presented on the upper coordinate axis and sex hormones index when the corresponding VOCs on the right longitudinal are axis fixed at the 25th, 50th, and 75th percentiles and the remaining metals are held at the 50th percentiles. Models adjusted for adjusts for age, race, education, marital status, body mass index (kg/m2), family income to poverty ratio, smoking and drinking.

We further constructed BKMR models to assess the combined effect of specific VOC exposure on sex hormones. For the sex hormones of testosterone, estradiol and SHBG, we found a positive overall association among the participants with above 50th percentile-level VOCs exposure, which was similar to each other. For the TT/E_2_, the overall negative association among the participants with above 50th percentile-level VOCs exposure. By contrast, multiple VOCs exposure showed a negative association with sex hormones, including testosterone, estradiol, SHBG and TT/E2 among those with below 50th percentile-level exposure (**Figure 6**). Meanwhile, we also investigated the exposure-response relationships between each of the seven VOCs and hormones when the exposure levels of the VOCs were at their representative 50th percentiles (**Figure 7**). We observed the positive nonlinear effects on blood 2,5-Dimethylfuran and testosterone, as well as estradiol. Negative nonlinear effects on blood Benzene and SHBG, as well as TT/E_2_, were also found. The interaction relationship between the eight specific VOCs and sex hormones was explored. We investigated the bivariate VOCs-response function of a single VOC, in which the second VOC was fixed at the 25th, 50th and 75th percentiles to see the interactions between every two VOCs. There was a potential interaction between blood Benzene and blood m-/p-Xylene in the SHBG model: the slope for blood Benzene decreased in the case of blood m-/p-Xylene, increasing from 25th to 75th (**Figure 8**). The other slopes of the bivariate VOCs-response function of certain VOCs were similar at different quantiles of another phthalate metabolite, with others fixed at their middle levels, which indicated no interactions.

### 3.7 Stratified associations between blood VOCs and sex hormones

We further ANALYZED stratified associations between blood VOCs and sex hormones in a specific subgroup by BMI shown in **Table 2**. Surprisingly, we found the association between the blood VOCs and the sex hormones concentrated in the specific subgroup among American adults. Testosterone increase by 277.712 (ng/dL) for each unit of Blood 2,5-Dimethylfuran, increase by 106.272 (ng/dL) for each unit of Blood Benzene, increase by 534.327 (ng/dL) for each unit of Blood Furan among the population whose BMI above 28 with statistical difference. Moreover, Testosterone decreased by -2.753 (ng/dL) for each unit of Blood 1,4-Dichlorobenzene, increased by 37.528 (ng/dL) for each unit of Blood Toluene and increase by 326.571 (ng/dL) for each unit of Blood Furan among the population whose BMI below 25. SHBG changed with various VOCs exposure in the specific subgroup. Population with BMI above 28 indicated that SHBG increase by 33.207 (ng/dL) for each unit of Blood Furan. Among the population whose BMI below 25, SHBG increase by 6.100 (ng/dL) for each unit of Blood Toluene and increased by 7.614 (ng/dL) for each unit of Blood m-/p-Xylene.

**Table 2:** Stratified associations of blood VOCs on sex hormones in the prespecified and exploratory subgroup.

Blood 2,5-Dimethylfuran	N	Testosterone	Estradiol	SHBG	Testosterone/Estradiol
Stratified by BMI					
≤25	774	248.161 (88.477, 407.846) 0.0024	8.324 (0.813, 15.835) 0.0302	19.021 (-2.153, 40.195) 0.0787	3.162 (-3.972, 10.296) 0.3852
25-28	625	120.792 (-53.911, 295.495) 0.1759	2.554 (-7.858, 12.966) 0.6308	21.031 (-3.017, 45.079) 0.0870	0.892 (-7.195, 8.979) 0.8289
>28	1242	**277.712 (167.753, 387.672) <0.0001**	3.784 (-3.303, 10.871) 0.2955	12.619 (-1.330, 26.569) 0.0765	5.500 (-1.838, 12.839) 0.1421
Blood Tetrachloroethene	N	Testosterone	Estradiol	SHBG	Testosterone/Estradiol
Stratified by BMI					
≤25	774	5.254 (-16.856, 27.364) 0.6415	-0.302 (-1.339, 0.735) 0.5680	0.155 (-2.766, 3.075) 0.9174	-0.104 (-1.086, 0.878) 0.8356
25-28	625	4.176 (-15.114, 23.466) 0.6715	-0.152 (-1.300, 0.996) 0.7953	1.847 (-0.806, 4.501) 0.1730	0.526 (-0.365, 1.417) 0.2477
>28	1242	7.608 (-9.016, 24.232) 0.3699	0.480 (-0.582, 1.541) 0.3759	0.184 (-1.907, 2.276) 0.8630	-0.317 (-1.417, 0.782) 0.5719
Blood Benzene	N	Testosterone	Estradiol	SHBG	Testosterone/Estradiol
Stratified by BMI					
≤25	774	-23.767 (-75.601, 28.066) 0.3691	-1.727 (-4.156, 0.702) 0.1639	-0.606 (-7.454, 6.243) 0.8624	-0.430 (-2.733, 1.874) 0.7149
25-28	625	8.808 (-74.091, 91.707) 0.8351	-0.141 (-5.075, 4.793) 0.9553	4.110 (-7.307, 15.527) 0.4807	0.871 (-2.960, 4.702) 0.6559
>28	1242	**106.272 (38.585, 173.960) 0.0021**	1.382 (-2.956, 5.719) 0.5325	5.655 (-2.885, 14.195) 0.1946	1.180 (-3.313, 5.674) 0.6067
Blood 1,4-Dichlorobenzene	N	Testosterone	Estradiol	SHBG	Testosterone/Estradiol
Stratified by BMI					
≤25	774	**-2.753 (-5.314, -0.192) 0.0355**	**-0.139 (-0.259, -0.019) 0.0234**	-0.330 (-0.668, 0.009) 0.0565	-0.044 (-0.158, 0.070) 0.4514
25-28	625	0.911 (-1.786, 3.608) 0.5082	0.047 (-0.113, 0.208) 0.5655	-0.071 (-0.443, 0.301) 0.7083	0.039 (-0.086, 0.163) 0.5421
>28	1242	1.253 (-0.340, 2.845) 0.1233	-0.021 (-0.123, 0.080) 0.6816	0.175 (-0.026, 0.375) 0.0875	-0.060 (-0.165, 0.046) 0.2663
Blood Ethl Acetate	N	Testosterone	Estradiol	SHBG	Testosterone/Estradiol
Stratified by BMI					
≤25	774	-1.659 (-5.324, 2.007) 0.3754	0.013 (-0.159, 0.185) 0.8864	0.027 (-0.457, 0.512) 0.9124	-0.098 (-0.261, 0.064) 0.2369
25-28	625	-5.940 (-14.035, 2.156) 0.1509	-0.305 (-0.787, 0.177) 0.2148	-0.676 (-1.792, 0.440) 0.2353	-0.183 (-0.558, 0.191) 0.3378
>28	1242	-2.285 (-6.028, 1.458) 0.2317	0.007 (-0.232, 0.247) 0.9515	0.006 (-0.465, 0.477) 0.9796	-0.127 (-0.374, 0.121) 0.3165
Blood Furan	N	Testosterone	Estradiol	SHBG	Testosterone/Estradiol
Stratified by BMI					
≤25	774	**326.571 (11.124, 642.019) 0.0428**	4.143 (-10.688, 18.975) 0.5842	33.408 (-8.296, 75.112) 0.1168	4.866 (-9.182, 18.913) 0.4974
25-28	625	260.056 (-61.475, 581.586) 0.1134	0.393 (-18.784, 19.570) 0.9680	34.551 (-9.754, 78.856) 0.1269	6.068 (-8.816, 20.952) 0.4245
>28	1242	**534.327 (305.695, 762.959) <0.0001**	2.288 (-12.432, 17.008) 0.7607	**33.207 (4.266, 62.148) 0.0247**	11.408 (-3.829, 26.644) 0.1425
Blood Toluene	N	Testosterone	Estradiol	SHBG	Testosterone/Estradiol
Stratified by BMI					
≤25	774	**37.528 (8.544, 66.512) 0.0114**	0.545 (-0.820, 1.909) 0.4343	**6.100 (2.280, 9.919) 0.0018**	-0.051 (-1.344, 1.242) 0.9381
25-28	625	3.290 (-19.239, 25.818) 0.7748	-0.564 (-1.904, 0.776) 0.4099	-0.272 (-3.376, 2.832) 0.8637	0.477 (-0.564, 1.518) 0.3693
>28	1242	5.267 (-7.596, 18.130) 0.4224	-0.322 (-1.143, 0.499) 0.4420	0.374 (-1.244, 1.992) 0.6503	0.022 (-0.829, 0.873) 0.9597
Blood m-/p-Xylene	N	Testosterone	Estradiol	SHBG	Testosterone/Estradiol
Stratified by BMI					
≤25	774	53.465 (-1.598, 108.528) 0.0574	-0.905 (-3.493, 1.683) 0.4932	**7.614 (0.345, 14.883) 0.0404**	0.012 (-2.440, 2.464) 0.9925
25-28	625	-2.973 (-17.777, 11.832) 0.6941	-0.279 (-1.160, 0.602) 0.5346	-0.285 (-2.325, 1.755) 0.7842	0.328 (-0.356, 1.011) 0.3482
>28	1242	3.607 (-8.008, 15.221) 0.5429	-0.357 (-1.098, 0.384) 0.3452	-0.447 (-1.908, 1.014) 0.5486	0.583 (-0.184, 1.351) 0.1367

Models adjusted age, race/ethnicity, education level, marital status, poverty to income ratio, Had at least 12 alcohol drinks/1 year, smoked at least 100 cigarettes in life.

Bold values indicated statistical difference with p values < 0.05.

### 3.8 Sensitivity analysis

In the sensitivity analysis, we refitted the models with urinary levels of mandelic acid and 2-methylhippuric acid and blood levels of 1,4-dichlorobenzene, 2,5-dimethylfuran, benzene, furan, tetrachloroethene, and toluene. The associations of the studied VOCs with sex hormones were similar to those of the main analysis (**Supplementary Figures 1**–**3**).

## 4 Discussion

To date, our research was the first to evaluate complex associations between Specific blood VOCs exposure and Sex Hormones in American adult males in the 2013-2014 and 2015-2016 NHANES study populations. Considering the large-scale sample size, we examined the independent and combined associations using multiple methods: the generalized linear models (GLM), XGBoost models, WQS regression analysis and BKMR analysis. Blood 2,5-dimethylfuran was found to be significantly and positively associated with testosterone and SHBG in multivariate weighted linear model analysis and had a marginal effect on Estradiol and TT/E2 after adjusting for the potential confounders. The findings of WQS regression suggested evident associations between co-exposure to all eight blood VOCs and increased testosterone, Estradiol and SHBG, which were mainly driven by Blood Benzene, Blood 1,4-Dichlorobenzene, Blood 2,5-Dimethylfuran and Blood Toluene. The univariate exposure-response function of the BKMR model revealed nonlinear relationships of Blood 2,5-Dimethylfuran, Blood Benzene, Blood Ethl Acetate with testosterone, Blood 2,5-Dimethylfuran and Blood Benzene with Estradiol and Blood Benzene and Blood Tetrachloroethene with SHBG. Furthermore, the bivariate exposure-response function suggested a potential interaction of Blood Ethl Acetate and Blood Benzene affecting TT/E2. The consistency of findings from traditional linear regressions and multipollutant effect analyses revealed that Blood 2,5-Dimethylfuran exposure might contribute to increased Male sex hormone.

Previous studies have shown that active smoking increases levels of different VOCs in the breath and blood (24, 25). Moreover, it has been reported that 2, 5-dimethylfuran is a highly selective biomarker of smoking status due to the compound’s ability to discriminate between social smokers and non-smokers (26). Moreover, studies have shown that smoking affects male sex hormones (27). And most studies on SHBG levels agree that smokers have higher SHBG levels (28, 29), wang etc (30) demonstrated that smokers had significantly higher total testosterone and free testosterone levels compared to nonsmokers. Therefore, the positive correlation between smoking and male sex hormones may be due to Blood 2,5-Dimethylfuran.

In our study, a nonlinear exposure–response relationship was found between blood Benzene concentrations and increased testosterone and Estradiol. Benzene is a chemical pollutant from natural and man-made sources, widely existing in the indoor and outdoor atmosphere (31). The Pearson correlation coefficient showed that a significant inverse correlation between the blood Benzene and testosterone (32), which is not consistent with our results. The possible reason is that this study is the first to examine the relationship between blood benzene and male sex hormones in humans exposed to natural conditions, while the above article is about occupational exposure. Importantly, we found Blood 2,5-Dimethylfuran in VOCs was the most significant effect on sex hormones in male. Testosterone increased by 213.594 (ng/dL) (124.552, 302.636) and estradiol increased by 7.229 (pg/mL) for each additional unit of blood 2,5-Dimethylfuran (ng/mL).

In industry, 1,4-Dichlorobenzene is used as a chemical intermediate for 1,2,4-trichlorobenzene, polyphenylene sulphide resin and dyes (33), 1, 4- Dichlorobenzene is prone to sublimation. Therefore, 1, 4- Dichlorobenzene is mainly exposed through inhalation (34). It is reported that exposure to 1, 4- Dichlorobenzene has been shown to increase white blood cell count and ALT activity (34). Moreover, research indicated that 1, 4- Dichlorobenzene may result in changes in endocrine functions and may affect the reproductive success of this and other species (35). However, there are no studies on the relationship between 1, 4- Dichlorobenzene and male sex hormones. We are the first study to suggest that 1, 4- Dichlorobenzene is negatively correlated with estradiol and TT/E_2_, with a large weight.

Also, our study has several advantages. Firstly, the individual and overall effects of blood VOCs exposure on male sex hormones were evaluated using WQS regression and the BKMR model, two methods for assessing the effects of multiple pollutants. In addition, the BKMR model was used to estimate the relationship between the nonlinear exposure effects and interactions of VOCs and male sex hormones. However, some limitations should also be noted. First, the study was based on data from the U.S. population, and there was a lack of data from other regions; Second, our study used a cross-sectional design, which limits causal inferences about the relationship between blood VOCs and male sex hormones studied. More prospective studies are needed to validate our findings. Third, VOCs have a relatively short half-life. There may be a time lag between male sex hormones and blood VOCs exposure. Forth, the weight factor was not taken into the BKMR model due to a lack of weight arguments, which may influence the extension of the result from the BKMR model. Finally, Residual confounding of unmeasured factors may bias our results to some extent.

## 5 Conclusion

In all, the WQS regression and XGBoost models were applied to find the relative important exposure factor to sex hormones among US adults. BKMR models reveal that Co-exposure to the VOCs was associated with increased TT, E2 and SHBG were the main contributors. GLM models revealed that specific VOC exposure as an independent risk factor might cause males metabolic disorders damage. We also identified the high-risk group on the VOCs exposure. Our research was a cross-sectional design. Further prospective and experimental studies are worth conducting to verify our findings and clarify the underlying biological mechanisms.

## Data availability statement

The original contributions presented in the study are included in the article/**Supplementary Material**. Further inquiries can be directed to the corresponding authors.

## Author contributions

CW: Conceptualization, Data curation, Formal analysis, Methodology, Software, Visualization, Writing – original draft, Writing – review & editing. LC: Conceptualization, Methodology, Writing – review & editing. YZ and WZ: Validation, Writing – review & editing. PZ and MW: Writing – review & editing. MX and CD: Writing – review & editing. QX and WL: Writing – review & editing. QH, YG, ZS, XC and ZC: Conceptualization, Funding acquisition, Methodology, Supervision, Writing – review & editing. All authors contributed to the article and approved the submitted version.
