# Supplementary figures and images for: Multiple statistical models reveal specific volatile organic compounds affect sex hormones in American adult male: NHANES 2013–2016
Source: Front Endocrinol (Lausanne). 2023 Jan 12;13:1076664. doi: 10.3389/fendo.2022.1076664 (PMC9877519; doi:10.3389/fendo.2022.1076664)

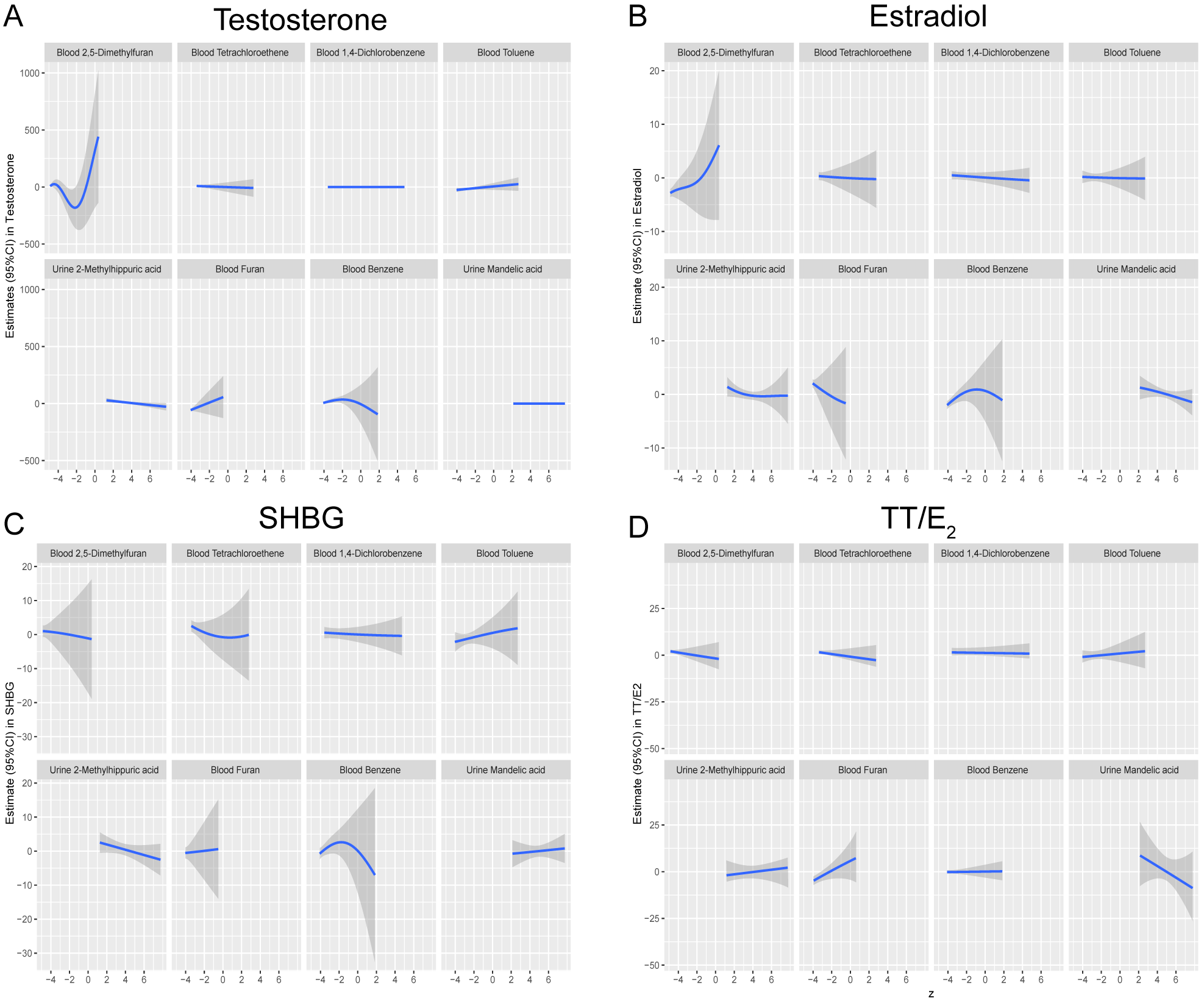

Supplement: Supplementary file 1 [file Image_1.tif]

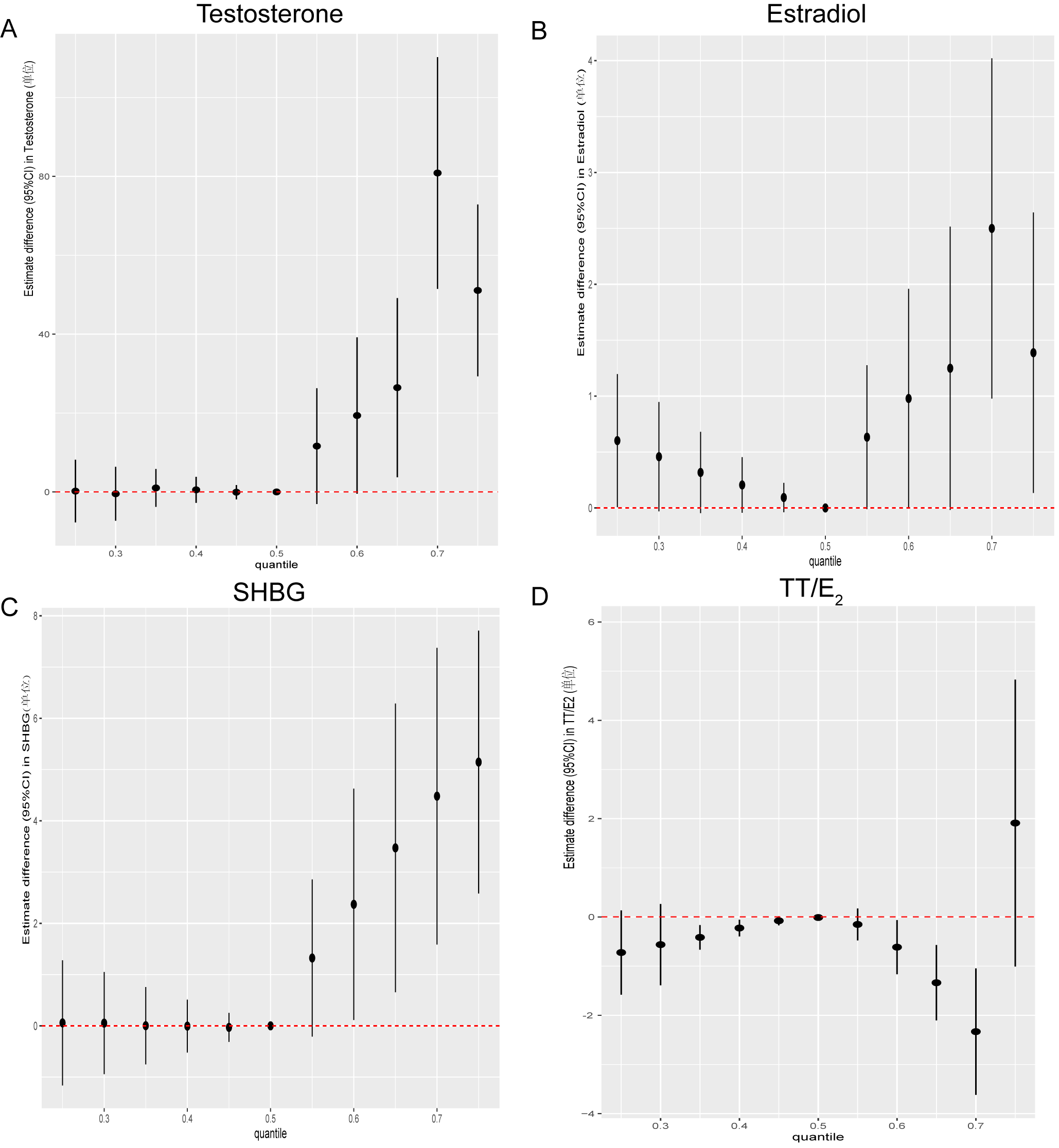

Supplement: Supplementary file 2 [file Image_2.tif]

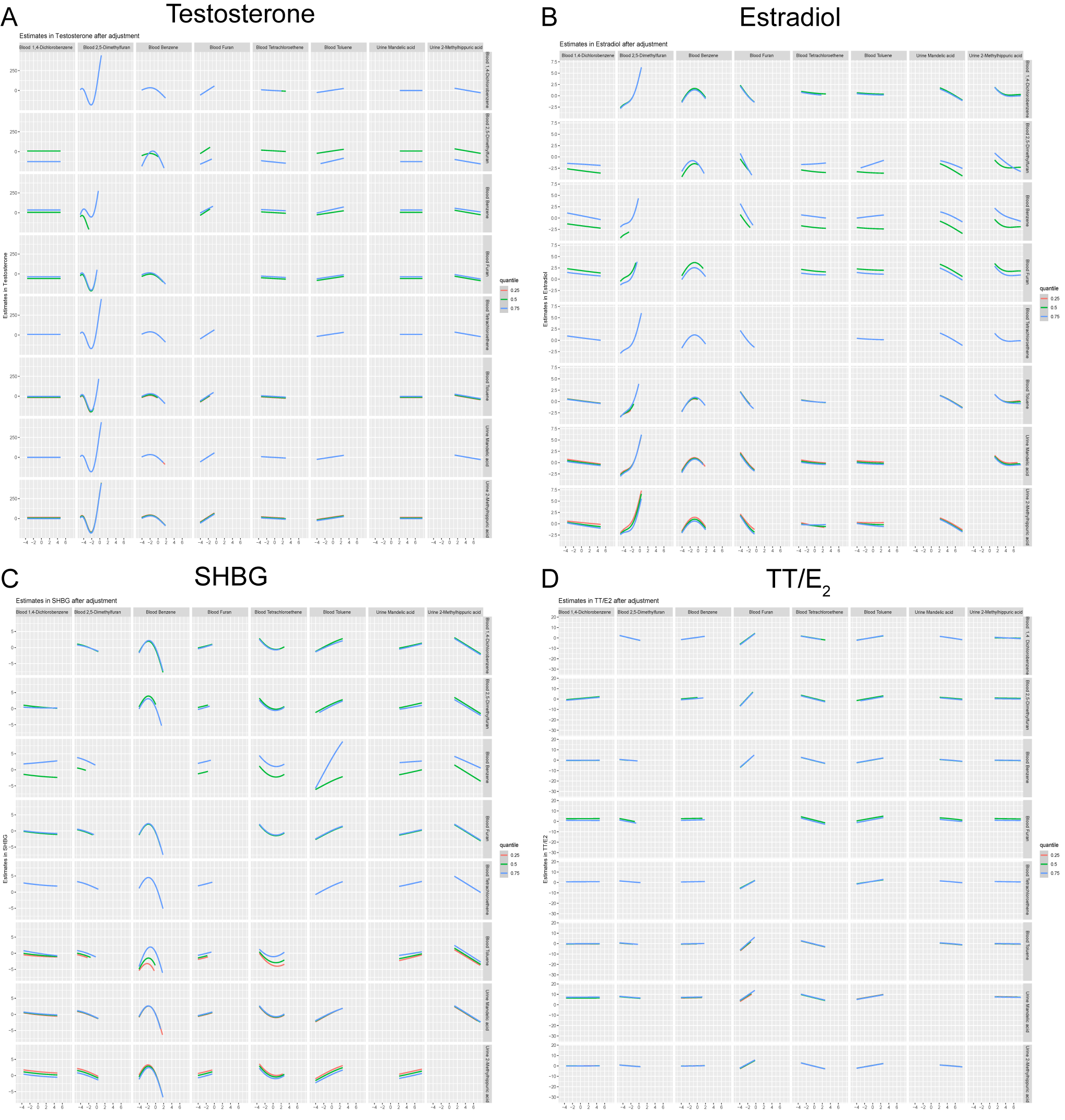

Supplement: Supplementary file 3 [file Image_3.tif]

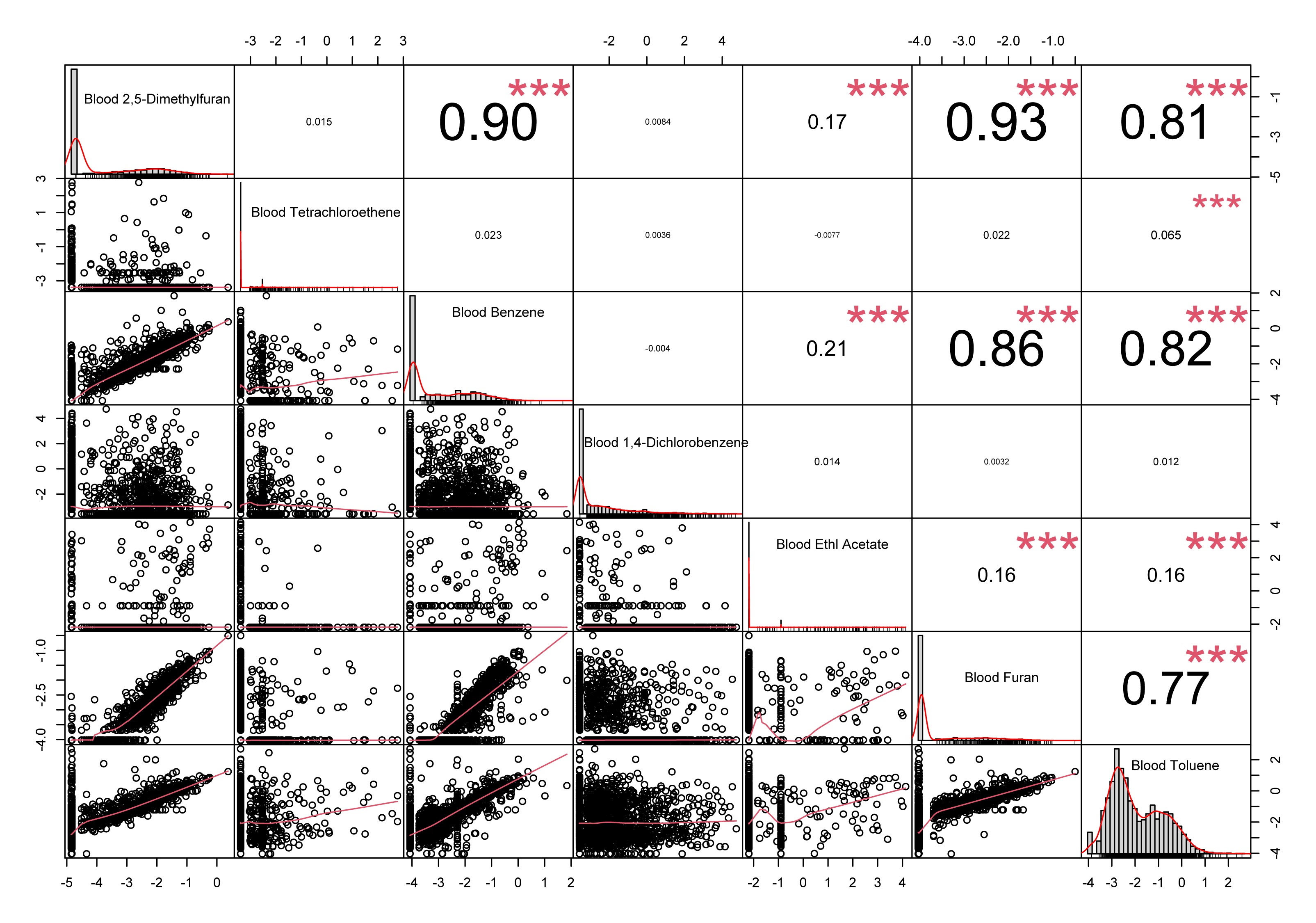

Supplement: Supplementary file 4 [file Image_4.tif]
